# Alliance of Efflux Pumps with β-Lactamases in Multidrug-Resistant *Klebsiella pneumoniae* Isolates

**DOI:** 10.1089/mdr.2018.0414

**Published:** 2019-10-11

**Authors:** Navdezda Maurya, Manoj Jangra, Rushikesh Tambat, Hemraj Nandanwar

**Affiliations:** Bioactive Screening & Clinical Microbiology Laboratory, CSIR-Institute of Microbial Technology, Chandigarh, India.

**Keywords:** *Klebsiella pneumoniae*, multidrug resistance, efflux pumps, extended spectrum β-lactamases

## Abstract

Nosocomial infections caused by *Klebsiella pneumoniae* are primarily characterized by a high prevalence of extended-spectrum β-lactamases (ESBL's) and a soaring pace of carbapenemase dissemination. Availability of limited antimicrobial agents as a therapeutic option for multidrug-resistant bacteria raises an alarming concern. This study aimed at the molecular characterization of multidrug-resistant *K. pneumoniae* clinical isolates and studied the role of efflux pumps in β-lactam resistance. Thirty-three isolates confirmed as ESBL-positive *K. pneumoniae* that harbored resistance genes to major classes of antibiotics. The results showed that CTX-M15 was the preeminent β-lactamase along with carbapenemases in ESBL-positive isolates. However, the efficacy of different antibiotics varied in the presence of lactamase inhibitors and efflux pump inhibitors (EPIs). Those showing increased efficacy of antibiotics with EPI were further explored for the expression of efflux pump genes and expressed a significantly different level of efflux pumps. We found that an isolate had higher expression of *kpnF* (SMR family) and *kdeA* (MATE family) pump genes relative to RND family pump genes. No mutations were observed in the genes for porins. Together, the findings suggest that β-lactamases are not the only single factor responsible for providing resistance against the existing β-lactam drugs. Resistance may increase many folds by simultaneous expression of RND family (the most prominent family in Gram-negative bacteria) and other efflux pump family.

## Background

*Klebsiella pneumoniae* imparts a significant burden on the health care system as a leading causative agent of nosocomial infections with multidrug resistance/extreme drug resistance (MDR/XDR) phenotype.^1^ The reduced efficacy of available drugs at a much faster pace is agitating the situation more than the nonavailability of new drugs.^2^ Among the few therapeutic options used as a last resort, one is carbapenems, from many β-lactam antibiotics approved. The increased use of carbapenems is also falling prey to the diversified carbapenemases, which make approximately half of the nosocomial strains of *K. pneumoniae* resistant.^3^ According to CDDEP, around 60% of Indian *K. pneumoniae* isolates are resistant to carbapenems and the use has been increased by ∼40% worldwide.^4^

Primary mechanisms of resistance against β-lactams are widely reported as enzymatic deactivation of antibiotics and reducing membrane permeability.^5^ Several reports provide evidence for the reduced uptake of antibiotics due to the permeability barriers caused by mutations or loss of porins.^6^ *K. pneumoniae* expresses four major porins LamB (48 kDa), OmpK36 (38 kDa), OmpK35 (36 kDa), and OmpK37. The OmpK36 and OmpK35 correspond to OmpF and OmpC of *Escherichia coli*.^7^ However, the role of efflux pumps in β-lactam resistance has remained skeptical for a long time, even though evident with the other classes of antibiotics.^8^

The resistance-nodulation-cell division (RND) systems, specially AcrAB, has been widely reported to generate MDR phenotype in Enterobacteriaceae.^9^ New efflux pumps of the RND family in *K. pneumoniae* continue to be described, for example, OqxAB (for quinolones)^10^ and KexD (for macrolides and tetracycline).^11^ Moreover, other efflux pumps such as KdeA of multidrug and toxic compound extrusion (MATE)^12^ and KpnEF of small multidrug resistance (SMR)^13^ family are also reported in *K. pneumoniae.*

Along with the identification of new drugs and drug targets, the effective study of drug resistance factors is also required. The contribution of efflux pumps is majorly stated on laboratory-generated bacterial strains; however, the studies on clinical isolates usually have attention on single efflux pump. Hence, the aim of this study is to analyze the contribution of other factors that is, efflux pumps/porins and concurrent expression of different efflux pumps under antibiotic stress.

## Methods

### Bacterial strains

Extended-spectrum β-lactamase (ESBL)-positive nosocomial isolates from ICU patients were collected during the year 2013–2014 from Government Medical College and Hospital (GMCH), Chandigarh, India. 16S rRNA gene sequences for all clinical isolates were amplified using primers 27F (5′-AGAGTTTGATCCTGGCTCAG-3′) and 1492R (5′-TACGGCTACCTTGTTACGACTT-3′)^14^ by conventional PCR and similarities were achieved using EzTaxon server.^*^ The taxonomically confirmed *K. pneumoniae* isolates were further taken for studies. *E. coli* ATCC 25922 was procured from MTCC, India. The remaining control strains were procured from Hi-media, India. The chemicals were purchased from Sigma Aldrich, or stated otherwise.

### Antibiotic susceptibility test

Antimicrobial susceptibility testing was performed by Kirby-Bauer disc diffusion method on Mueller-Hinton agar (MHA) and zone of inhibitions (ZOI) were measured and interpreted as per recommendations by Clinical and Laboratory Standards Institute, 2019 (CLSI).^15,16^ Antibiotic discs used in the study were procured from Hi-media Laboratories, India. Susceptibility of isolates to different classes of antibiotics, that is, penicillins [ampicillin (10 μg), methicillin (5 μg), and piperacillin (100 μg)], β-lactam/β-lactamase inhibitor combination [amoxicillin/clavulanic acid (20 μg/10 μg), ampicillin/sulbactam (10 μg/10 μg), and piperacillin/tazobactam (100 μg/10 μg)], cephems [cefixime (5 μg), ceftazidime (30 μg), cefotaxime (30 μg), ceftriaxone (30 μg), and cefuroxime (30 μg)], monobactams [aztreonam (30 μg)], macrolide [azithromycin (15 μg), clarithromycin (15 μg,) and erythromycin (15 μg)], aminoglycoside [streptomycin (10 μg), gentamicin (120 μg), neomycin (30 μg), kanamycin (30 μg), and amikacin (30 μg)], tetracycline (30 μg), fluoroquinolones [ciprofloxacin (5 μg), gemifloxacin (5 μg), and norfloxacin (10 μg)], and folate pathway inhibitor (trimethoprim (5 μg), fosfomycin (200 μg), and nitrofurantoin (300 μg)), was checked.

### Phenotypic detection of β-lactamase production and efflux pump activity

The production of ESBL was assessed in clinically isolated *K. pneumoniae* by microdilution assay following the CLSI guidelines.^16^ Briefly, minimum inhibitory concentration (MIC) in the presence of β-lactamase inhibitor (potassium clavulanate) was determined. The isolates with fold reduction of ≥4 in the MIC of aztreonam, cefotaxime, ceftriaxone, and ceftazidime in the presence of clavulanic acid were considered ESBL producers. For phenotypic detection of efflux pump activity, MIC of antimicrobial agents, either alone or in the presence of efflux pump inhibitors (EPIs) (carbonyl cyanide-chlorophenylhydrazone, CCCP, and phenylalanine-arginine β-naphthylamide, PAβN), was determined for aztreonam, ceftazidime, and imipenem as representative of monobactam, third-generation cephalosporins, and carbapenem antibiotic family, respectively.

Microdilution assay in accordance with CLSI recommendation was followed with cation-adjusted Mueller-Hinton Broth (caMHB) (BD™ Difco™).^16^ The MIC for β-lactamase inhibitor and EPIs ranged from 64–125 μg/mL to 125–500 μg/mL, respectively. The subinhibitory concentration of β-lactamase inhibitor (16 μg/mL), CCCP (16 μg/mL), and PAβN (25 μg/mL) was used in synergy assays. Cells (10^5^ cfu/mL) were incubated at 37°C for 18 hrs in 96-well flat-bottom plates and growth was observed by the addition of 20 μL of 3-(4,5-dimethylthiazol-2-yl)-2,5-diphenyltetrazolium bromide (MTT, 10 mg/mL). MIC determination was performed in duplicate.

### Detection of β-lactamase genes

The genomic DNA and the plasmids were isolated using Quick-DNATM Fungal/Bacterial Miniprep Kit (Zymo-Research, Irvine, CA) and Plasmid Miniprep Kit (Qiagen^®^, Hilden, Germany), respectively. Presence of *bla_SHV_*, *bla_TEM_*, *bla_KPC_*, and *bla_CTX-M_* group (*bla_CTX-M1_*, *bla_CTX-M2_*, *bla_CTX-M3_*, and *bla_CTX-M4_*), *bla_NDM_*, *bla_VIM_*, *bla_IMP_*, and *bla_OXA_* genes (Table 1), was detected by PCR. Following PCR, sequencing of the PCR products was performed and compared with reported sequences from Genbank. *K. pneumoniae* ATCC 700603 (ESBL positive), *K. pneumoniae* ATCC BAA-2146 (NDM positive), *K. pneumoniae* ATCC BAA-1705 (KPC positive)*, K. pneumoniae* ATCC BAA-1706 (KPC negative), and *E. coli* ATCC 25922 (ESBL negative) were used as controls in this study.

**Table 1.** Oligonucleotides for the Detection of β-Lactamase Genes

*Primers*	*Sequence (5′-3′)*	*Reference/Accession No.*
bla_SHV_-F	GGGTTATTCTTATTTGTCGC	^32^
bla_SHV_ -R	TTAGCGTTGCCAGTGCTC	
bla_TEM_-F	ATAAAATTCTTGAAGACGAAA	^32^
bla_TEM_-R	GACAGTTACCAATGCTTAATCA	
bla_CTX-M_-1F	GACGATGTCACTGGCTGAGC	^33^
bla_CTX-M_-1R	AGCCGCCGACGCTAATACA	
bla_CTX-M_-2F	GCGACCTGGTTAACTAACAATCC	^33^
bla_CTX-M_-2R	CGGTAGTATTGCCCTTAAGCC	
bla_CTX-M_-3F	CGCTTTGCCATGTGCAGCACC	^33^
bla_CTX-M_-3R	GCTCAGTACGATCGAGCC	
bla_CTX-M_-4F	GCTGGAGAAAAGCAGCGGAG	^33^
bla_CTX-M_-4R	GTAAGCTGACGCAACGTCTG	
bla_KPC_-F	TGTCTTGTCTCTCATGGCCG	NC_014312.1
bla_KPC_-R	TTACTGCCCGTTGACGCCCAA	
bla_IMP_-F	AGCAGAGCCTTTGCCAGATT	^34^
bla_IMP_-F	TGATGCGTCTCCAGCTTCAC	
bla_VIM_-F	GTGCTTTGACAACGTTCGCT	^34^
bla_VIM_-F	AAGTCCGTTAGCCCATTCCG	
bla_NDM_-F	CCCGGTCGCGAAGCTGAGC	NC_023908.1
bla_NDM_-R	TCAGCGCAGCTTGTCGGCCA	
bla_OXA_-F	ATTATCGGAATGCCTGCGGT	NC_019154.1
bla_OXA_-F	AAACCATCCGATGTGGGCAT	
ompK35-F	ATGATGAAGCGCACTATTCTGG	NC_016845.1
ompK35-R	CATGACGAGGTTCCATTGTG	
ompK35p-F	CCTTTACCCGCACATCTTGC	NC_016845.1
ompk35p-R	CGATACGGGCATAGGTGGTATCGTC	
ompK36-F	ATGAAAGTTAAAGTACTGTCCCTCCTGG	HM000046.1
ompK36-R	GAACTGGTAAACCAGGCCCA	
ompK36p-F	TGCCGCCCAGGAATTATCTT	HM000046.1
ompk36p-R	CCAGGAGGGACAGTACTTTAACTTTCAT	

### Expression analysis of efflux pump genes

Expression of efflux pump genes was studied in clinical isolates showing ≥8-fold reduction (FR) in MIC with either of the EPIs. The culture was grown in caMHB and incubated in the presence or absence of subinhibitory concentrations (1/10th of MIC) of antibiotics at 10^5^ cfu/mL. Total RNA was isolated from isolates after 4 hrs of incubation using TRIzol method in an RNase-free environment. The purity and concentration of extracted RNA were assessed by nanodrop using Synergy™ H1 hybrid multimode reader (BioTek, Winooski, VT) at 260/280 nm and adjusted to 150 ng/mL. The real-time quantification of RNA templates was performed using SYBR green one-step RT-PCR kit (Invitrogen^®^) on Applied Biosystems^®^ 7500 Real-Time PCR. The data were analyzed using the 2^−ΔΔCt^ method and normalized against the expression of *K. pneumoniae rpoB* gene. All the experiments were performed in duplicates with two biological repeats. The primers used in this study are listed in Table 2.

**Table 2.** Oligonucleotides for the Detection of Efflux Pump Genes in RT-PCR

*Primers*	*Sequence (5′-3′)*	*Reference/Accession No.*
rpoA-F	AAGGCGAATCCAGCTTGTTCAGC	^35^
rpoA-R	TGACGTTGCATGTTCGCACCCATCA	
acrB-F	GTCGGTACAGGCGTAATGGG	AJ318073.1
acrB-R	TAGCGGCCTTTTGTTCAGGA	
oqxA-F	CGCGTCTCGGGATACATTGA	CP025456.1
oqxA-R	AATAGGGGCGGTCACTTTGG	
kdeA-F	CCATACTACGCCGGTCAACA	CP003999.1
kdeA-R	CCCGTTATGTCTGGTGCTGT	
kexD-F	AGCGTACTGGCCGTATGATG	CP003999.1
kexD-R	TCCTTGAGGGTGGTGAATGC	
kpnF-F	AAAGATTGCCCTCGGTGTGG	NC_012731.1
kpnF-R	TCAGCAGGGACAGACTCTCA	

### Multilocus sequence typing

Multilocus sequence typing (MLST) of the cultures was performed for seven housekeeping genes: *gapA*, *infB*, *mdh*, *pgi*, *phoE*, *rpoB*, and *tonB*. Sequence types were determined using the MLST database.^17,**^

### Outer membrane protein and Porin isolation

Outer membrane proteins (OMPs) were extracted by rapid microprocedure for outer membranes as described elsewhere with minor modifications.^18^ Briefly, overnight grown cultures in Nutrient broth (supplemented with 20% sorbitol to mimic the *in vivo* conditions) were pelleted and washed with 10 mM HEPES buffer (pH 7.4) followed by cell lysis by sonication (two cycles of 30 sec, each comprising 6 × 5 sec). The lysate was initially centrifuged at 15,600 *g* for 2 min to remove the intact cells and followed by centrifugation at 15,600 *g* for 30 min. The pellet was resuspended in 0.2 mL of 10 mM HEPES buffer (pH 7.4) and an equal volume of 2% sodium lauroyl sarcosinate in 10 mM HEPES buffer (pH 7.4) was added to solubilize membrane proteins and incubated for 30 min at room temperature. Insoluble OMPs were pelleted again as described above and washed with 0.5 mL of 10 mM HEPES buffer (pH 7.4), and then solubilized in 50 μL of 10 mM HEPES buffer (pH 7.4). Electrophoretic analysis of OMPs was performed in polyacrylamide gels with 4% stacking and a 12% separating gel. OMPs were detected with Coomassie brilliant blue R-250 staining.

## Results

### Antimicrobial susceptibility

**FIG. 1.**
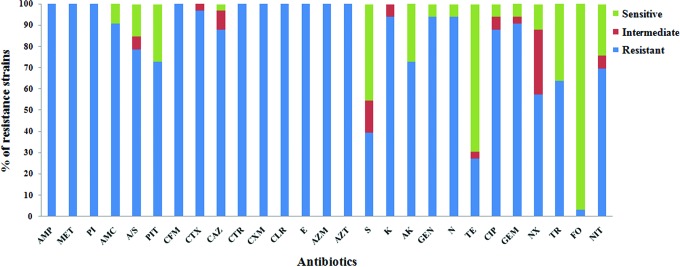
Antibiotic susceptibility test of 33 ESBL-positive clinical isolates. AMP, Ampicillin; MET, Methicillin; PI, Piperacillin; AMC, Amoxyclav; A/S, Ampicillin/sulbactam; PIT, Piperacillin/Tazobactam; CFM, Cefixime; CTX, Cefotaxime; CAZ, Ceftazidime; CTR, Ceftriaxone; CXM, Cefuroxime; CLR, Clarithromycin; E, Erythromycin; AZM, Azithromycin; AZT, Aztreonam; S, Streptomycin; K, Kanamycin; AK, Amikacin; GEN, Gentamicin; N, Neomycin; TE, Tetracycline; CIP, Ciprofloxacin; GEM, Gemifloxacin; NX, Norfloxacin; TR, Trimethoprim; FO, Fosfomycin; NIT, Nitrofurantoin. Color images are available online.

Seventy-eight nosocomial isolates were obtained; among them, thirty three were confirmed as ESBL-positive *K. pneumoniae*. All further studies were carried out with these ESBL-positive isolates. In this population, one-third of the strains (33.3%) exhibited XDR phenotype (resistant to one antimicrobial agent in all, but one or two antimicrobial class) and rest (66.6%) showed MDR phenotype (resistance to at least one antibiotic in three or more antimicrobial class). More than 90% of the isolates were resistant to macrolides, fluoroquinolones, glycopeptides, and β-lactams along with inhibitors. However, the isolates were susceptible to fosfomycin (96.9%) and to some extent to tetracycline (69.6%) (Fig. 1).

### β-lactamase identification

Genomic and plasmid DNA were isolated from the strains to check the presence or absence of β-lactamases by PCR. The total population (33 ESBL-positive *K. pneumoniae*) contained *bla_CTX-M-15_* gene (100%) and among them, 75.75% (25 isolates) exhibited carbapenemase genes. From the isolates with carbapenemase gene, 23 contained *bla_NDM_* (69.6%) and 8 were found to have additional carbapenemase genes as well (5 isolates with *bla_IMP_* and 3 isolates with *bla_OXA-181_*) (Table 3). We observed no amplification for *bla_KPC_* gene in any of the isolates.

**Table 3.** Characterization of Gene Sequences Along with Fold Reduction in MIC Values for Different Antibiotics with β-Lactam Inhibitor and Efflux Pump Inhibitors Among ESBL-Positive *Klebsiella pneumoniae* Isolates

*Isolates*	*β-lactamases*	*Carbapenemase*	*AZT+CA*	*AZT+EP1*	*AZT+EP2*	*CAZ+CA*	*CAZ+EP1*	*CAZ+EP2*	*IM+CA*	*IM+EP1*	*IM+EP2*
GMCH01	SHV, CTX-M15	NDM	16	1	2	1	1	1	4	4	1
GMCH02	TEM, CTX-M15	NDM	64	4	2	1	1	8	8	4	1
GMCH03	SHV, CTX-M15	OXA-181, NDM	512	32	8	1	2	2	1	1	1
GMCH04	SHV, CTX-M15	OXA-181, NDM	256	256	16	1	2	2	1	1	1
GMCH07	SHV, TEM, CTX-M15	—	32	2	2	1	2	1	1	2	1
GMCH08	SHV-1, TEM, CTX-M15	NDM	64	1	1	1	1	1	16	4	2
GMCH09	SHV-28,TEM, CTX-M15	NDM	128	2	4	1	2	2	1	2	1
GMCH10	SHV-28, TEM, CTX-M15	IMP, NDM	1	128	2	1	8	2	1	64	2
GMCH11	SHV, TEM, CTX-M15	NDM	2000	1	2	2	1	4	16	8	4
GMCH12	SHV, TEM, CTX-M15	NDM, IMP	256	1	1	8	1	2	50	16	8
GMCH13	SHV, TEM, CTX-M15	NDM, IMP	4	1	1	32	1	1	4	4	4
GMCH15	SHV, TEM, CTX-M15	OXA-181	1	1	4	1	1	2	4	4	4
GMCH16	SHV, TEM, CTX-M15	NDM	16	16	16	128	2	2	16	1	1
GMCH19	SHV, TEM, CTX-M15	—	8	4	2	32	4	4	1	1	1
GMCH20	SHV, CTX-M15	OXA-181, NDM,	128	2	4	256	1	4	2	2	2
GMCH1101	SHV, TEM, CTX-M15	—	2053	2	2	512	2	8	16	4	2
GMCH1249	SHV, TEM, CTX-M15	NDM	4	2	8	2	2	4	32	4	2
GMCH1144	SHV, TEM, CTX-M15	NDM	128	1	1	64	1	2	8	4	4
GMCH1143	SHV, TEM, CTX-M15	NDM	8	1	1	4	1	2	4	2	1
GMCH490	SHV, TEM, CTX-M15	—	128	1	2	128	2	2	4	2	1
GMCH827	SHV, CTX-M15	IMP, NDM	32	8	1	253	2	2	16	8	2
GMCH1428	SHV, TEM, CTX-M15	—	128	8	4	4	2	1	64	4	1
GMCHB8	SHV-55, TEM	NDM	64	1	2	2	1	2	128	1	1
GMCH1573	CTX-M15	NDM	128	0.5	4	128	1	1	1	1	1
GMCH976	SHV, TEM, CTX-M15	NDM	256	8	16	1	2	4	8	8	2
GMCH1522	SHV, CTX-M15	IMP, NDM	8	4	2	8	1	1	2	1	1
GMCH1243	CTX-M15	—	128	1	1	1	1	1	0.5	1	0.5
GMCH8941	SHV, TEM, CTX-M15	IMP	32	2	1	64	2	2	8	4	2
GMCH14543	CTX-M15	—	16	2	1	8	16	1	8	4	1
GMCH7708	CTX-M15	—	32	8	2	8	8	1	4	8	1
GMCH14114	CTX-M15	NDM	32	1	4	32	1	1	8	1	1
GMCH8006	TEM, CTX-M15	NDM	128	2	1	16	1	1	32	8	4
GMCH7662	CTX-M15	NDM	513	2	4	128	2	4	64	16	8

The MIC fold reduction ≥8-fold is highlighted in gray.

AZM, Aztreonam; CA, potassium clavulanic acid (16 μg/mL); CAZ, ceftazidime; IM, imipenem; EP1, CCCP (16 μg/mL); EP2, PAβN (25 μg/mL).

### Minimum inhibitory concentration

All the clinical isolates, when analyzed for MIC, exhibited high resistance against aztreonam and ceftazidime. For imipenem (≥ 4 μg/mL), 90.9% (30 isolates) were resistant (Table 3 and Supplementary Table S1). It was observed that potassium clavulanate increased the antimicrobial activity of the antibiotics and reduced the MIC at the subinhibitory concentration. The change in MICs of antibiotics in the presence of EPI is one of the most widely used phenotypic methods to check the role of the efflux pumps. The FR in MICs caused by EPI may be attributable to the affinity of the EPI to the efflux pump, with the expression level of the efflux pumps or with interference with the efflux pump energy source.^19^ In the presence of EPIs (CCCP or/and PAβN), maximum FR was observed only in nine isolates (27.27%) with the aztreonam. Furthermore, with ceftazidime, five, and with imipenem, only seven isolates showed significant FR.

Remaining isolates displayed no significant change in MIC in the presence of either of the inhibitors. The highest FR was observed in isolate GMCH04 (256-fold with CCCP and 16-fold with PAβN) followed by GMCH10 (128-fold with CCCP and twofold with PAβN). GMCH10 also showed the highest FR with imipenem (64-fold). In the case of ceftazidime, FR of 8 was observed with GMCH02 and GMCH1101. Likewise, GMCH12 and GMCH7662 showed 8-FR in MIC when PAβN was co-administered with imipenem (Table 3).

### Efflux pump expression

**FIG. 2.**
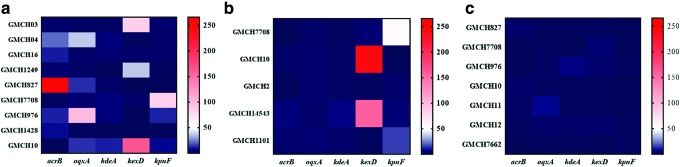
Heat map for the relative expression of *acrB*, *oqxA*, *kexD*, *kdeA*, and *kpnF* with antibiotics. Clinical isolates with ≥8-fold reduction with efflux pump inhibitors were analyzed for expression. Clinical isolates were incubated with or without EPI at the respective subinhibitory concentration of different antibiotics. Six hours later, mRNA expressions were analyzed through qRT-PCR. Expressions were normalized against *rpoB* gene and represented as a heat map with **(a)** aztreonam, **(b)** ceftazidime, and **(c)** imipenem. Relative expressions are displayed in *dark* to *light* color as low to high expressions, respectively. EPI, efflux pump inhibitor. Color images are available online.

The isolates with ≥8-FR in MIC with EPIs were further analyzed for expression levels of efflux pump genes: *acrB*, *oqxA*, *kexD*, *kdeA*, and *kpnF*, with or without the antibiotic (Table 3). In the presence of a subinhibitory concentration of aztreonam and ceftazidime, RND efflux pump gene expression was observed to be upregulated in most of the isolates. Some isolates showed higher expression of *oqxA* (GMCH04 and GMCH976 for aztreonam; and GMCH02 for ceftazidime) and *kexD* (GMCH03 and GMCH1249 for aztreonam; and GMCH10 and GMCH14543 for ceftazidime) compared to *acrB* of the RND family (Supplementary Table S2). However, in GMCH7708, the expression of SMR pump gene *kpnF* (83.20 ± 57.2-fold) was higher, followed by *kdeA* (5.41 ± 7.6-fold) with aztreonam (Fig. 2a). Furthermore, the isolate also showed higher expression of *kpnF* (63.41 ± 43.4-fold) with ceftazidime (Fig. 2b) and marginal expression with imipenem (Fig. 2c).

However, the expression of efflux pumps in the presence of imipenem was observed to be the lowest. The isolate GMCH976, showing 97.85 ± 23.8-fold expression of *oqxA* and 12.0 ± 9.5-fold of *kexD* with aztreonam, showed <10-fold expression (5.99 ± 3.6-fold: *kdeA* & 2.27 ± 2.7-fold: *kexD*) with imipenem (Fig. 2c and Supplementary Table S2). The simultaneous expression of efflux pumps (either of the same class or different class) may have an additive or synergistic effect on resistance. Few isolates were observed with high expression of efflux pumps from other families; however, resistance was not as high compared to the isolates with higher RND pump expression. Furthermore, the MLST suggested that these isolates belonged to diverse sequence types (ST) with ST15 being the most common (7 isolates) (Supplementary Table S2).

### Porin mutation and gene expression

**FIG. 3.**
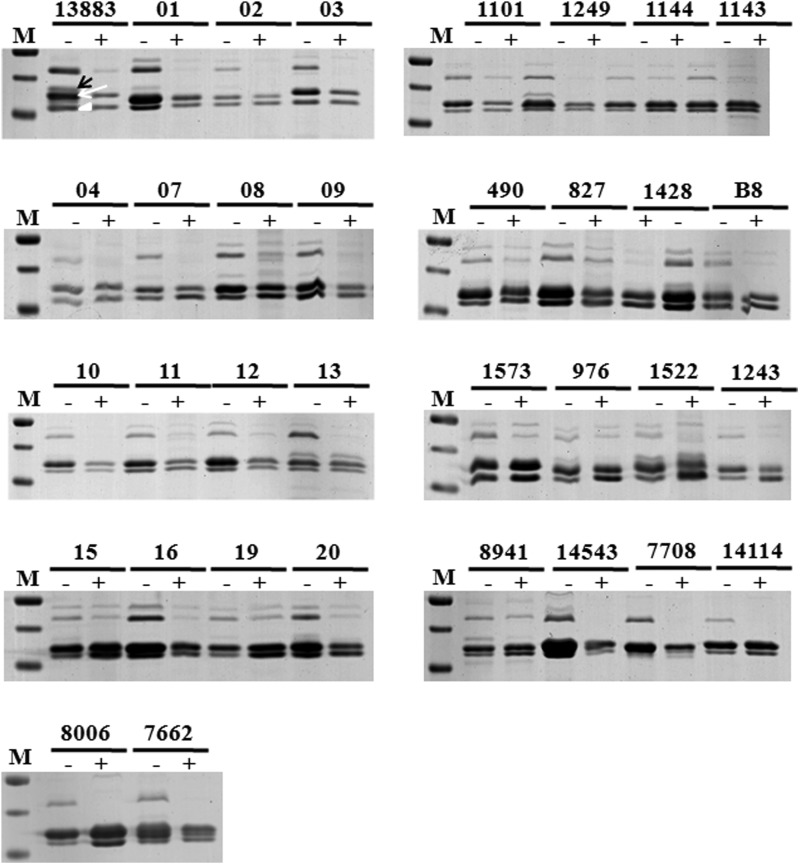
SDS-PAGE analysis of OMPs of *Klebsiella pneumoniae* isolates. M: Marker; C: ATCC 13883; 1-7662: *K. pneumoniae* isolates. The SDS-PAGE analysis of OMPs extracted from *K. pneumoniae* isolates in nutrient broth (−) or nutrient broth with 20% sorbitol (+) overnight. M: Protein molecular weight marker; ATCC13883: control; 1-7662: *K. pneumoniae* isolates. The 12% SDS-PAGE was performed with 20 μg of isolated outer membrane proteins with 6 M urea. The band identified as OmpK36 by trypsin digestion following MALDI-TOF/MS-MS analysis is marked by a *white arrow*, and the position of OmpK35 and OmpA band is also indicated (*black arrow* and *arrowhead*, respectively). OMP, outer membrane protein; SDS-PAGE, sodium dodecyl sulfate-polyacrylamide gel electrophoresis.

The role of porin deficiency is not solely responsible for imparting high resistance; however, it always accompanies with the other factors. Despite various reports supporting the loss of porins along with the high-level production of ESBLs or AmpC β-lactamases, we were unable to detect any mutations in the major porin genes (Fig. 3). The absence of OmpK35 in the lower osmolarity media may be due to factors such as the mutation in the repressor or overexpression of the transcriptional regulators. Furthermore, no differences were found in the promoter regions (TTGCAC-35 box and TACAAT-10 box) and sequences of OmpK35 and OmpK36 among *K. pneumoniae* ATCC 13883 and various clinical isolates.

## Discussion

The clinical significance of efflux pumps toward β-lactams is being actively explored for the last few decades in Enterobacteriaceae. The identification and characterization of MDR efflux pumps in clinical settings are required to design a course of treatment. Hence, we studied the role of efflux pumps in β-lactam-resistant clinical isolates. During the study, most of the selected clinical isolates exhibited co-resistance with aminoglycosides, glycopeptides, fluoroquinolones, and tetracyclines. The co-presence of these resistance genes limits the therapeutic options during the treatment, pushing the use of last-line drugs. Similar results have been observed in the study of Enterobacteriaceae clinical isolated strains from north India as they co-expressed β-lactamases with other genes conferring resistance to aminoglycosides, macrolide, rifampicin, and sulfamethoxazole.^20^

Among β-lactamases, CTX-M type β-lactamases is the most frequent ESBL worldwide.^21^ We also observed the presence of bla_CTX-M15_ gene in the isolates and were consistent with the published reports from India. CTX-M-15, CTX-M-16, and CTX-M-19 are reported to hydrolyze ceftazidime efficiently,^22^ which partially explains the high MIC observed for ceftazidime against the tested isolates. NDM (which accounts for the majority of carbapenem resistance) was found to be the next resistance-causative genes after bla_CTX-M-15._ Carbapenem resistance in *K. pneumoniae* is reported to have increased from 29% in 2008 to 57% in 2014^4^ and has been linked to VIM, KPC, OXA, and NDM-1.^23,24^ However, in this study, very few isolates harbored OXA-181 and IMP. In addition, the presence of metallo-β-lactamase (MBL) raised the MIC for imipenem. It has been shown that the contemporaneous presence of ESBL and MBL results in the high MIC of ceftazidime along with other β-lactams.^25^ This supports similar findings in this study as well.

Besides their substantial roles (*e.g*., virulence, homeostasis, and maintaining intracellular trafficking), the magnitude of the clinical significance of multidrug efflux pumps is escalating.^26^ The expression of different efflux pumps varies according to different antibiotic stress, which can be reflected in the isolates' MICs to different β-lactam antibiotics. The restoration of β-lactam activity observed in the presence of EPI indicates the role of efflux systems in tested clinical isolates, similar to various other antibiotic families like chloramphenicol, tetracyclines, and quinolones. The activity of an EPI is influenced by its uptake in the outer membrane and periplasmic concentration. Moreover, some EPIs can be more selective for a specific efflux pump. Thus, the selectivity/efficacy of efflux pump is directly connected with the activity of the respective EPI and on the degree of altered resistance.^5^

Generally expressed at a basal level, higher expression of these pumps can be achieved either constitutive (by mutations) or transient (under specific conditions), both resulting in antibiotic resistance. Several reports indicate majorly the significance of AcrAB pump in MDR bacteria,^9,2^ but the isolates such as GMCH03, GMCH1249, and GMCH976 were observed with higher expressions of other RND efflux pump genes like *kexD*. Similarly, above 90-fold upregulation of *acrB* genes in MDR *K. pneumoniae* has been reported beside *oqxB*.^27^ In another study, significant upregulation of *oqxB* expression than *acrB* is reported in tigecycline-resistant *K. pneumoniae*.^1^ This signifies the clinical importance of other RND efflux pumps.

Furthermore, the isolates GMCH7708, GMCH10, and GMCH976 showed the significant expressions of *kdeA* and *kpnF* components along with RND family components. The FR in MIC in the presence of EPI and higher expression of different efflux pump genes advocate synergistic/additive effect of these pumps in the efflux of β-lactam family. Furthermore, the FR in MIC determined for imipenem in KexD expressing *E. coli* and *K. pneumoniae*,^11^ and for cephalosporins in KpnEF expressing *K. pneumoniae*^13^ in two independent studies also support the role of these pumps in β-lactam resistance. The properties of different antibiotics or substrates of efflux pumps also influence the expression level. Substrates with more hydrophobicity and large molecular weight (300–600 Da) are preferably effluxed out compared to lipophilic/zwitterionic and small molecular weight (<300 Da).^28^ This explains the significant upregulation of efflux pumps under aztreonam (435.433 Da) and ceftazidime (546.58 Da), while imipenem (299.347 Da) produces no such effect.

It has been shown previously that porin loss, but not pump overproduction, raises the MIC values for imipenem.^29^ The isolates may lack the expression of large diffusion channels and express a smaller channel porin.^30^ All the isolates grown under low osmolarity conditions showed prominent expression of OmpK36 and OmpA porins, whereas, on increasing osmolarity, the OmpK36 expression was reduced. OmpK35 porin expression was observed in a few isolates and was absent in high osmolarity. Numerous reports show the involvement of the porins in assisting resistance; however, unlike the presence of β-lactamase, the porin deficiency by itself cannot raise the MICs considerably.^31^

The resistance mechanism in clinical isolates is diverse and we need to understand the molecular basis of these convoluted factors. To conclude, among the different mechanisms deployed by bacteria to acquire resistance against the β-lactams, efflux pump plays no exception toward them. The synergistic/additive effect of different efflux pumps provides an advantage to nosocomial agents against antibiotic combination therapies. Furthermore, the small fractions of isolates were capable of overexpressing efflux pumps (such as MATE and SMR) other than RND family, but at present, not contributing much to resistance. Hence, the resistance may revamp with the use of EPIs in combinatorial therapy.

## Ethics Approval and Consent to Participate

The use of clinical isolates was approved by the institutional biosafety committee (IBSC no: IMTECH/IBSC/2015/08) of the Institute of Microbial Technology, India.

## Availability of Data and Materials

All data generated or analyzed during this study are included in this published article.

## Supplementary Material

Supplemental data

Supplemental data

## Footnotes

^*^ http://eztaxon-e.ezbiocloud.net/

^**^ www.pasteur.fr/mlst
