# Supplementary material for: Alliance of Efflux Pumps with β-Lactamases in Multidrug-Resistant Klebsiella pneumoniae Isolates
Source: Microb Drug Resist. 2019 Oct 11;25(8):1155–63. doi: 10.1089/mdr.2018.0414 (PMC6807647; doi:10.1089/mdr.2018.0414)
Supplement: Supplemental data [file Supp_TableS1.pdf]

Supplementary Data

Supplementary Table S1. MIC Values Observed for Different Antibiotics with β-Lactam Inhibitor and Efflux Pump Inhibitors Among ESBL-Positive *K. pneumoniae* Isolates

Strains	*AZT*	*AZT+CA*	*AZT+EP1*	*AZT+EP2*	*CAZ*	*CAZ+CA*	*CAZ+EP1*	*CAZ+EP2*	*IM*	*IM+CA*	*IM+EP1*	*IM+EP2*
GMCH01	500	31.25	500	250	>4,000	4,000	>4,000	4,000	64	16	16	64
GMCH02	1,000	15.78	250	500	>4,000	4,000	>4,000	500	64	8	16	64
GMCH03	>2,000	3.9	62.5	250	>4,000	>4,000	>2,000	2,000	>128	>128	>128	>128
GMCH04	>2,000	7.8	7.8	125	>4,000	>4,000	>2,000	2,000	>128	128	>128	>128
GMCH07	500	15.8	250	250	>4,000	4,000	>2,000	>4,000	16	16	8	16
GMCH08	500	7.8	500	500	>4,000	>4,000	>4,000	>4,000	>128	8	32	64
GMCH09	500	3.9	250	125	>4,000	>4,000	>2,000	2,000	16	16	8	16
GMCH10	62.5	62.5	0.487	31.25	250	250	31.25	125	64	64	1	32
GMCH11	500	0.250	500	250	4,000	2,000	4,000	1,000	16	1	2	4
GMCH12	>2,000	7.8	2,000	2,000	4,000	500	4,000	2,000	16	0.315	1	2
GMCH13	>2,000	500	2,000	2,000	>4,000	125	>4,000	4,000	64	16	16	16
GMCH15	125	125	125	31.25	4,000	4,000	4,000	2,000	64	16	16	16
GMCH16	31.25	1.9	1.9	1.9	>4,000	31.25	>2,000	2,000	8	0.5	8	8
GMCH19	500	62.5	125	250	>4,000	125	>1,000	1,000	8	8	8	8
GMCH20	1,000	7.8	500	250	4,000	15.6	4,000	1,000	8	4	4	4
GMCH1101	1,000	0.487	500	500	500	0.975	250	62.5	2	0.125	0.5	1
GMCH1249	250	62.5	125	31.25	250	125	125	62.5	8	0.250	2	4
GMCH1144	2,000	15.62	2,000	2,000	1,000	15.62	1,000	500	8	1	2	2
GMCH1143	2,000	250	2,000	2,000	1,000	250	1,000	500	4	1	2	4
GMCH490	2,000	15.6	2,000	1,000	1,000	7.8	500	500	4	1	2	4
GMCH827	250	7.9	31.25	250	4,000	15.8	2,000	2,000	8	0.500	1	4
GMCH1428	1,000	7.8	125	250	4,000	1,000	2,000	4,000	128	2	32	128
GMCHB8	1,000	15.62	1,000	500	>4,000	2,000	>4,000	2,000	64	0.5	64	64
GMCH1573	1,000	7.8	2,000	250	>4,000	31.25	>4,000	4,000	>128	>128	>128	>128
GMCH976	2,000	7.8	250	125	4,000	4,000	2,000	1,000	8	1	1	4
GMCH1522	2,000	250	500	1,000	4,000	500	4,000	4,000	16	8	16	16
GMCH1243	500	3.9	500	500	4,000	4,000	>4,000	4,000	16	32	16	32
GMCH8941	125	3.9	62.5	125	125	1.95	62.5	62.5	8	1	2	4
GMCH14543	125	7.8	62.5	125	125	15.8	7.8	125	1	0.125	0.25	1
GMCH7708	250	7.9	31.25	125	62.5	7.9	7.8	62.5	1	0.25	0.125	1
GMCH14114	500	15.6	500	125	250	7.8	250	250	8	1	8	8
GMCH8006	250	1.95	125	250	125	7.8	125	125	8	0.25	1	2
GMCH7662	250	0.487	125	62.5	62.5	0.487	31.25	15.62	4	0.062	0.25	0.5

The isolates with ≥8-fold reduction with either EPI are highlighted in gray.
AZM, Aztreonam; CA, Potassium clavulanic acid; CAZ, Ceftazidime; IM, Imipenem; EP1, CCCP (16 μg/mL); EP2, PAβN (25 μg/mL).
