# Supplementary material for: Alliance of Efflux Pumps with β-Lactamases in Multidrug-Resistant Klebsiella pneumoniae Isolates
Source: Microb Drug Resist. 2019 Oct 11;25(8):1155–63. doi: 10.1089/mdr.2018.0414 (PMC6807647; doi:10.1089/mdr.2018.0414)
Supplement: Supplemental data [file Supp_TableS2.pdf]

Supplementary Table S2. The Relative Expression of *acrB*, *oqxA*, *kexD*, *kdeA*, and *kpnF* with Antibiotics for Clinical Isolates with ≥8-Fold Reduction

		Aztreonam					*Ceftazidime*					*Imipenem*				
Isolates	*ST**	*AcrB*	*OqxA*	*KdeA*	*KexD*	*KpnF*	*AcrB*	*OqxA*	*KdeA*	*KexD*	*KpnF*	*AcrB*	*OqxA*	*KdeA*	*KexD*	*KpnF*
GMCH03	ST147	2.29 ± 0.78	1.22 ± 0.62	1.19 ± 0.48	84.22 ± 57.14	2.95 ± 0.96	ND	ND	ND	ND	ND	ND	ND	ND	ND	ND
GMCH04	ST147	21.46 ± 19.22	37.32 ± 37.25	5.26 ± 4.8	0.64 ± 1.2	1.92 ± 2.18	ND	ND	ND	ND	ND	ND	ND	ND	ND	ND
GMCH16	ST134	11.05 ± 1.7	1.79 ± 1.37	3.23 ± 3.12	1.83 ± 2.3	1.96 ± 0.7	ND	ND	ND	ND	ND	ND	ND	ND	ND	ND
GMCH1249	ST2837	2.88 ± 0.6	1.48 ± 0.4	0.15 ± 0.06	36.85 ± 50.29	0.75 ± 0.77	ND	ND	ND	ND	ND	ND	ND	ND	ND	ND
GMCH827	ST437	265.64 ± 210	13.58 ± 10.29	1.61 ± 1.9	0.26 ± 1.02	0.07 ± 2.11	ND	ND	ND	ND	ND	2.66 ± 0.92	0.43 ± 0.09	0.88 ± 0.71	0.71 ± 0.12	0.22 ± 0.004
GMCH7708	ST15	1.05 ± 1.3	3.32 ± 2.9	5.41 ± 7.6	1.78 ± 2.4	83.20 ± 57.21	0.19 ± 0.3	0.26 ± 03..7	0.509 ± 0.78	3.09 ± 1.3.6	63.43 ± 43.4	0.05 ± 0.03	0.70 ± 0.77	0.06 ± 0.02	3.87 ± 1.26	0.40 ± 0.08
GMCH976	ST15	11.79 ± 328.46	97.85 ± 23.86	5.26 ± 6.58	0.15 ± 0.2	12.0 ± 9.56	ND	ND	ND	ND	ND	0.27 ± 0.01	1.23 ± 1.42	5.99 ± 3.64	2.27 ± 2.7	1.07 ± 0.27
GMCH1428	ST15	8.46 ± 8.8	0.58 ± 0.29	0.39 ± 0.25	0.09 ± 0.1	0.45 ± 0.18	ND	ND	ND	ND	ND	ND	ND	ND	ND	ND
GMCH10	ST15	0.27 ± 0.12	14.09 ± 8.77	11.54 ± 2.17	164.29 ± 28.03	7.9 ± 2.11	1.78 ± 0.42	2.58 ± 1.46	1.59 ± 0.26	242.14 ± 73.7	0.15 ± 0.11	1.7 ± 1.23	1.06 ± 0.74	0.80 ± 0.43	0.44 ± 0.48	0.07 ± 0.09
GMCH02	ST15	ND	ND	ND	ND	ND	0.55 ± 0.09	3.12 ± 1.45	1.18 ± 0.26	0.90 ± 0.72	2.07 ± 1.1	ND	ND	ND	ND	ND
GMCH14543	ST147	ND	ND	ND	ND	ND	4.94 ± 0.83	2.05 ± 2.7	6.20 ± 0.69	156.85 ± 93.36	4.15 ± 1.02	ND	ND	ND	ND	ND
GMCH1101	ST23	ND	ND	ND	ND	ND	2.91 ± 1.07	3.53 ± 2.42	2.43 ± 1.11	0.73 ± 0.33	15.54 ± 4.39	ND	ND	ND	ND	ND
GMCH11	ST15	ND	ND	ND	ND	ND	ND	ND	ND	ND	ND	1.51 ± 0.34	2.27 ± 0.48	0.48 ± 0.19	0.70 ± 0.14	0.37 ± 0.11
GMCH12	ST437	ND	ND	ND	ND	ND	ND	ND	ND	ND	ND	0.89 ± 0.34	0.03 ± 1.59	1.59 ± 1.71	2.25 ± 2.81	1.08 ± 1.12
GMCH7662	ST15	ND	ND	ND	ND	ND	ND	ND	ND	ND	ND	0.23 ± 0.002	0.24 ± 0.59	0.59 ± 0.02	0.19 ± 0.11	1.16 ± 0.48

*The ST represents the Sequence Types of isolates obtained from MLST.
ND, not determined.
